# Transmembrane Protein Oxygen Content and Compartmentalization of Cells

**DOI:** 10.1371/journal.pone.0002726

**Published:** 2008-07-16

**Authors:** Rajkumar Sasidharan, Andrew Smith, Mark Gerstein

**Affiliations:** 1 Molecular Biophysics and Biochemistry Department, Yale University, New Haven, Connecticut, United States of America; 2 Department of Computer Science, Yale University, New Haven, Connecticut, United States of America; 3 Interdepartmental Program in Computational Biology and Bioinformatics, Yale University, New Haven, Connecticut, United States of America; Utrecht University, Netherlands

## Abstract

Recently, there was a report that explored the oxygen content of transmembrane proteins over macroevolutionary time scales where the authors observed a correlation between the geological time of appearance of compartmentalized cells with atmospheric oxygen concentration. The authors predicted, characterized and correlated the differences in the structure and composition of transmembrane proteins from the three kingdoms of life with atmospheric oxygen concentrations in geological timescale. They hypothesized that transmembrane proteins in ancient taxa were selectively excluding oxygen and as this constraint relaxed over time with increase in the levels of atmospheric oxygen the size and number of communication-related transmembrane proteins increased. In summary, they concluded that compartmentalized and non-compartmentalized cells can be distinguished by how oxygen is partitioned at the proteome level. They derived this conclusion from an analysis of 19 taxa. We extended their analysis on a larger sample of taxa comprising 309 eubacterial, 34 archaeal, and 30 eukaryotic complete proteomes and observed that one can not absolutely separate the two groups of cells based on partition of oxygen in their membrane proteins. In addition, the origin of compartmentalized cells is likely to have been driven by an innovation than happened 2700 million years ago in the membrane composition of cells that led to the evolution of endocytosis and exocytosis rather than due to the rise in concentration of atmospheric oxygen.

## Introduction

The evolution of multicellular forms of life represents a major transition in the evolution of complex organisms. Cellular compartmentalization was a natural consequence of this evolutionary shift that lead to a large-scale innovation in the origin of protein domains with roles in cellular communication. However, the events that led to this shift are not clear. On a sample size of 19 proteomes that comprised 6 eukaryotes, 9 eubacteria and 4 archaea, Acquisti et al. observed a correlation between the time of appearance of cellular compartmentalization and atmospheric oxygen concentration[1]. They propose that atmospheric oxygen levels influenced the composition of transmembrane proteins, and that older taxa (bacteria and archaea) exclude oxygen from low-oxygen transmembrane proteins to a greater extent than do younger taxa. They suggest that this constraint relaxed over time when atmospheric oxygen concentrations rose and led to the increase in size and number of receptors. They hypothesized that atmospheric oxygen concentrations affected the timing of evolution of cellular compartmentalization by constraining the size of domains necessary for cellular communication.

## Results and Discussion

**Figure 1:**
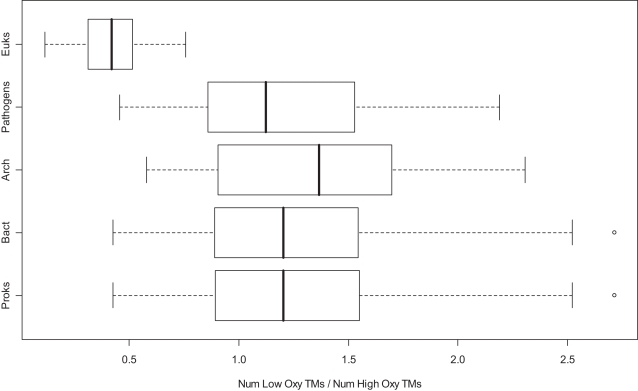
Partition of oxygen in different membrane proteomes. Boxplot of ratio of the number of low oxygen transmembrane proteins to the number of high-oxygen transmembrane proteins for 373 complete genomes (30 eukaryote, 34 archaea and 309 eubacteria). We removed four outliers in prokaryotes that had a ratio greater than 3. Key: Euks–Eukaryotes, Arch–Archaeabacteria, Bact–Eubacteria, and Proks–Prokaryotes.

To support their claims the authors calculate the ratio of the number of low-oxygen transmembrane proteins to the number of high-oxygen ones for these 19 proteomes (Figure 5a on Page 50 of their article) and suggest that single-celled compartmentalized organisms, namely *Saccharomyces cerevisiae*, *Giardia lamblia* (unicellular eukaryotes), and *Rhodopirellula baltica* (planctomycete bacteria), are similar to eukaryotes in the proportion of their proteome that are high-oxygen transmembrane proteins. Eubacteria and archaea are similar in this respect and the authors infer from this that there is no systematic association between eukaryotic complexity, proteome oxygen partitioning, and atmospheric oxygen levels. They claimed this boils down to a basic functional difference associated with proteome composition and state that, of the 19 taxa, “*the only two groups that can be distinguished by how oxygen is partitioned at the proteome level are compartmentalized and non-compartmentalized cells*”. In our work, we reproduced this figure on a larger sample of complete proteomes (309 eubacteria, 34 archaea, and 30 eukaryote) available from NCBI's ftp site[2] (Figure 1a) using the same methods that authors used to derive their results. We observed that ∼13% of the prokaryotic proteomes (40 eubacteria and 4 archaea excluding multiple strains) exhibit a ratio that is less than the highest ratio of 0.76 observed for a eukaryote (*Bos Taurus*). About 50% of the eukaryotes overlap with the range that we observed for prokaryotes. While there is a trend for lower ratios in eukaryotic proteomes, our data indicate that one cannot find an absolute separation of compartmentalized and non-compartmentalized groups of organisms based solely on how oxygen is partitioned among their transmembrane proteins. Thus, the genomes that the authors used in their analysis are likely to be biased and as our data indicate the key conclusion they derive is not true on a larger sample of genomes.

It has been well-established the ‘great oxidation event’ that resulted in the shift from an anoxic to an oxic atmosphere occurred about 2.0 billion years ago[3]. Molecular fossils derived from cellular and membrane lipids provide convincing evidence that eukaryotes first appeared before 2.7 billion years[4]. Thus, primitive forms of compartmentalized eukaryotes are likely to have evolved in an anoxic atmosphere where oxygen concentration was between 0.001 and 0.01% of the present atmospheric level of oxygen (21%)[5]. These compartmentalized eukaryotic forms had flexible outer membranes due to the inclusion of sterols that allowed them to ingest prokaryotes leading to the creation of organelles in the form of new compartments[6], [7]. Sterols are not found in Archaea and their occurrences in Bacteria are sparsely distributed. However, they are ubiquitous features of eukaryotic membranes and their biosynthetic pathway is very highly conserved within eukaryotic domains. It also appears likely that the initial steps in sterol biosynthesis were present in their modern form in the last common ancestor of eukaryotes[7]. The functional equivalent of sterols in Bacteria is the hopanoids whose biosynthetic pathway is highly conserved within prokaryotes. However, the fundamental difference between these two classes of molecules is that membrane deformation is achieved much more readily by sterol-containing membranes than hopanoids or any membrane lipid. Thus, a membrane with a well-regulated sterol composition becomes a useful tool for export and import across the cell membrane[7]. The innovation of sterol biosynthesis in much less atmospheric oxic conditions, in allowing rapid membrane deformation, may have been a key step in the evolution of endocytosis and exocytosis and thus, cellular compartmentalization leading to other advances like signaling by the Eukaryotes.

Most transmembrane domains in Eukaryotes[8] and *R.baltica* [9] are novel kingdom-specific and species-specific protein domains, respectively, that have roles in cell communication, cell adhesion and cell differentiation. While these domains exhibit higher oxygen content, it is not clear what functional roles the oxygen-containing amino acids may have. Also, most cytoplasmic domains in Eukaryotes and Prokaryotes are enriched for higher oxygen content, and these domains function in a particularly reducing environment. Furthermore, the absence of Eubacterial orthologs for Eukaryotic transmembrane domains makes a general comparison of the two sets of transmembrane domains unmatched. Given these, there simply may not have been any selective pressure to increase the oxygen content in the transmembrane domains of Eubacteria.

## Methods

We downloaded fasta files of predicted protein sets of complete genomes (309 eubacteria, 34 archaea, and 30 eukaryote) from NCBI's ftp server. We wrote a PERL script to calculate the mean oxygen content of a protein sequence and used the software TMHMM[10] to predict transmembrane protein topology for the complete proteomes.
